# WRN Nuclease‐Mediated EcDNA Clearance Enhances Antitumor Therapy in Conjunction with Trehalose Dimycolate/Mesoporous Silica Nanoparticles

**DOI:** 10.1002/advs.202407026

**Published:** 2024-08-29

**Authors:** Yinan Li, Xiu Huang, Yingying Li, Qingqing Qiao, Caihong Chen, Yang Chen, Weilong Zhong, Huijuan Liu, Tao Sun

**Affiliations:** ^1^ State Key Laboratory of Medicinal Chemical Biology and College of Pharmacy Nankai University Tianjin 300350 China; ^2^ Tianjin Key Laboratory of Digestive Diseases Department of Gastroenterology and Hepatology Tianjin Institute of Digestive Diseases Tianjin Medical University General Hospital Tianjin 300052 China; ^3^ Tianjin Key Laboratory of Early Druggability Evaluation of Innovative Drugs Tianjin Key Laboratory of Molecular Drug Research Tianjin International Joint Academy of Biomedicine Tianjin 300450 China

**Keywords:** extrachromosomal DNA, granuloma, scarring, trehalose dimycolate, tumor fibrosis

## Abstract

Current research on tumor fibrosis has focused on cancer‐associated fibroblasts, which may exert dual functions of tumor promotion and inhibition. Little attention has been paid to whether tumor cells themselves can undergo fibrotic transformation and whether they can inhibit parenchymal cells similar to pulmonary fibrosis, thus achieving the goal of inhibiting the malignant progression of tumors. To explore the significance of inducing tumor fibrosis for cancer treatment. This study utilizes mesoporous silica nanoparticles (MSN) loaded with Trehalose dimycolate (TDM) to induce tumor cell fibrosis through the dual effects of TDM‐induced inflammatory granuloma and MSN‐induced foreign body granuloma. The results show that TDM/MSN (TM) can effectively induce tumor fibrosis, manifested specifically by collagen internalization, and suppression of proliferation and invasion capabilities, suggesting the potential role of tumor fibrosis therapy. However, further investigation reveals that extrachromosomal DNA (ecDNA) mediates resistance to fibrosis induction. To comprehensively enhance the efficacy, WRN exonuclease is conjugated to TM to form new nanoparticles (TMW) capable of effectively eliminating ecDNA, globally promoting tumor cell fibroblast‐like transformation, and validated in a PDX model to inhibit cancer progression. Therefore, TMW, through inducing tumor cell fibrosis to inhibit its malignant progression, holds great potential as a clinical treatment strategy.

## Graphical abstract

This study demonstrates that mesoporous silica nanoparticles (MSN) loaded with Trehalose dimycolate (TDM) induce tumor cell fibrosis through inflammatory and foreign body granulomas, effectively suppressing tumor proliferation and invasion. WRN exonuclease conjugation to TDM/MSN further enhances fibrosis by eliminating ecDNA, offering a promising strategy for cancer treatment.

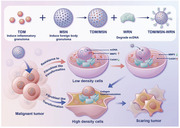

## 1 Introduction

Current drug therapies for tumors primarily rely on chemotherapy, targeted therapy, and immunotherapy. However, there is currently no research on the development of new therapies aimed at inducing tumor fibrosis by mimicking organ fibrosis. Fibrosis is characterized by the formation of a dense network of collagen fibers and represents a crucial mechanism of body tissue repair mode.^[^ ^1^ ^]^ Current research suggests that tumor fibrosis plays a dual role in tumor growth and metastasis. Tumor fibrosis typically represents the process of extracellular matrix (ECM) remodeling in the tumor microenvironment, with the main ECM components including collagen proteins (primarily type I and type III), elastin, and proteoglycans.^[^ ^2^ ^]^ It is currently believed that tumor fibrosis is driven by stromal components, with research on this process mainly focusing on cancer‐associated fibroblasts (CAFs). The role of CAFs within the tumor microenvironment (TME) is still a matter of controversy. Many attempts to target CAFs in anti‐tumor research have yielded contradictory results, and some clinical studies have shown that high expression of CAFs markers in the tumor microenvironment is favorable for prognosis.^[^ ^3^ ^]^ The uncertain role of CAFs in the TME may be attributed to their diverse cellular origins, including resident fibroblasts within tissues, adipocytes within tumor tissues, and pericytes.^[^ ^4^ ^]^ However, rarely attention has been given to whether tumor cells themselves undergo fibrotic transformation and the potential biological effects it may bring. Tumor cells are widely recognized as highly plastic, capable of undergoing Epithelial‐Mesenchymal Transition (EMT) to acquire an interstitial cell phenotype, characterized by enhanced migratory and invasive abilities.^[^ ^5^ ^]^ The formation of scar tissue begins with granulation tissue and progresses through organization to gradually form fibrosis.^[^ ^6^ ^]^ During this process, fibroblasts enter a quiescent state. Therefore, inducing tumor cells to undergo fibroblastic transformation may enable them to acquire the ability to secrete collagen, leading to organization and ultimately resulting in stiff state. This process could potentially achieve the suppression of malignant tumor progression.

Bacillus Calmette‐Guérin (BCG) is an attenuated vaccine derived from Mycobacterium bovis and was initially developed as a vaccine against tuberculosis. BCG has been proven to be the first successful immunotherapy and has been used for the treatment of various cancers for many years.^[^ ^7^ ^]^ It remains a frontline adjuvant therapy for bladder cancer, effectively reducing cancer recurrence and preventing its spread. However, the therapeutic efficacy of BCG is inconsistent, and it faces challenges in penetrating solid tumors. It also carries potential life‐threatening side effects such as systemic bacteremia and significant organ damage.^[^ ^8^ ^]^ Therefore, our research also focuses on how to mitigate these risks and enhance its anti‐tumor effects by leveraging the properties of nanomedicine. Trehalose dimycolate (TDM) is the most abundant hydrophobic glycolipid on the cell wall of BCG. It has been reported to possess anti‐tumor properties and various immune‐stimulating activities.^[^ ^9^ ^]^ By binding to Macrophage‐Inducible C‐Type Lectin (Mincle), TDM triggers inflammation, mediates infective granuloma formation, and subsequently leads to fibrosis.^[^ ^10^ ^]^ Research on the anti‐tumor effects of TDM primarily focuses on immunomodulatory, such as activating dendritic cells and CD8^+^ T cells to induce anti‐tumor immunity.^[^ ^9^ ^]^ However, the impact of TDM on tumor cells themselves and its ability to induce fibroblast‐like transformation in tumor cells remains unclear. Furthermore, the low water solubility of TDM limits its application as a drug, making it difficult to deliver into the interior of solid tumors. Mesoporous silica nanoparticles (MSN) have attracted considerable attention in nanomedicine delivery due to their tunable pore size, high pore volume, large surface area ensuring high drug loading capacity, ease of fabrication, and unique potential for immune stimulation.^[^ ^11^ ^]^ Moreover, as a drug carrier, MSN can also exploit the enhanced permeability and retention (EPR) effect to achieve retention in tumor tissues.^[^ ^12^ ^]^ Therefore, MSN holds promise as a carrier for TDM to form new nanoparticles for anti‐tumor effects. Additionally, silicon dioxide can hydrolyze in tumor tissues to generate orthosilicic acid.^[^ ^13^ ^]^ Upon hydration, it mediates macrophage aggregation, leading to enhanced antigenicity. Then triggers a foreign body granulomatous pathological effect similar to “silicosis nodules” leading to tumor fibrosis.^[^ ^14^ ^]^

In this study, we intend to develop a new anti‐tumor strategy by preparing novel nanoparticles of MSN‐loaded TDM, utilizing the dual‐induced fibrotic effects of MSN and TDM, and constructing a mouse subcutaneous loaded tumor model to observe the anti‐tumor effects and the possibility of inhibiting the malignant progression of tumors by inducing the fibroblastic transformation of tumor cells. The combined in vitro and in vivo studies indicate that TM can effectively enter solid tumors and inhibit tumor growth and metastasis by inducing a non‐EMT fibroblast‐like transformation and internal accumulation of collagen in tumor cells. However, it was observed that some cells exhibited resistance to fibroblastic differentiation due to the elevated levels of extrachromosomal DNA (ecDNA). Therefore, further coupling of the nucleotide enzyme WRN on TM helped in clearing ecDNA, enhancing the efficacy of inducing tumor fibrosis. This study reveals the potential of TDM/MSN‐WRN (TMW) on inducing tumor cells to undergo fibroblast‐like transformation and the associated intracellular collagen accumulation, which can inhibit the malignant progression of tumors. It provides a novel anti‐tumor paradigm and offers new theoretical foundations and strategies for designing anti‐tumor drugs.

## 2 Results

### 2.1 Characterization and Antitumor Evaluation of MSN Loaded with TDM

**Figure 1:**
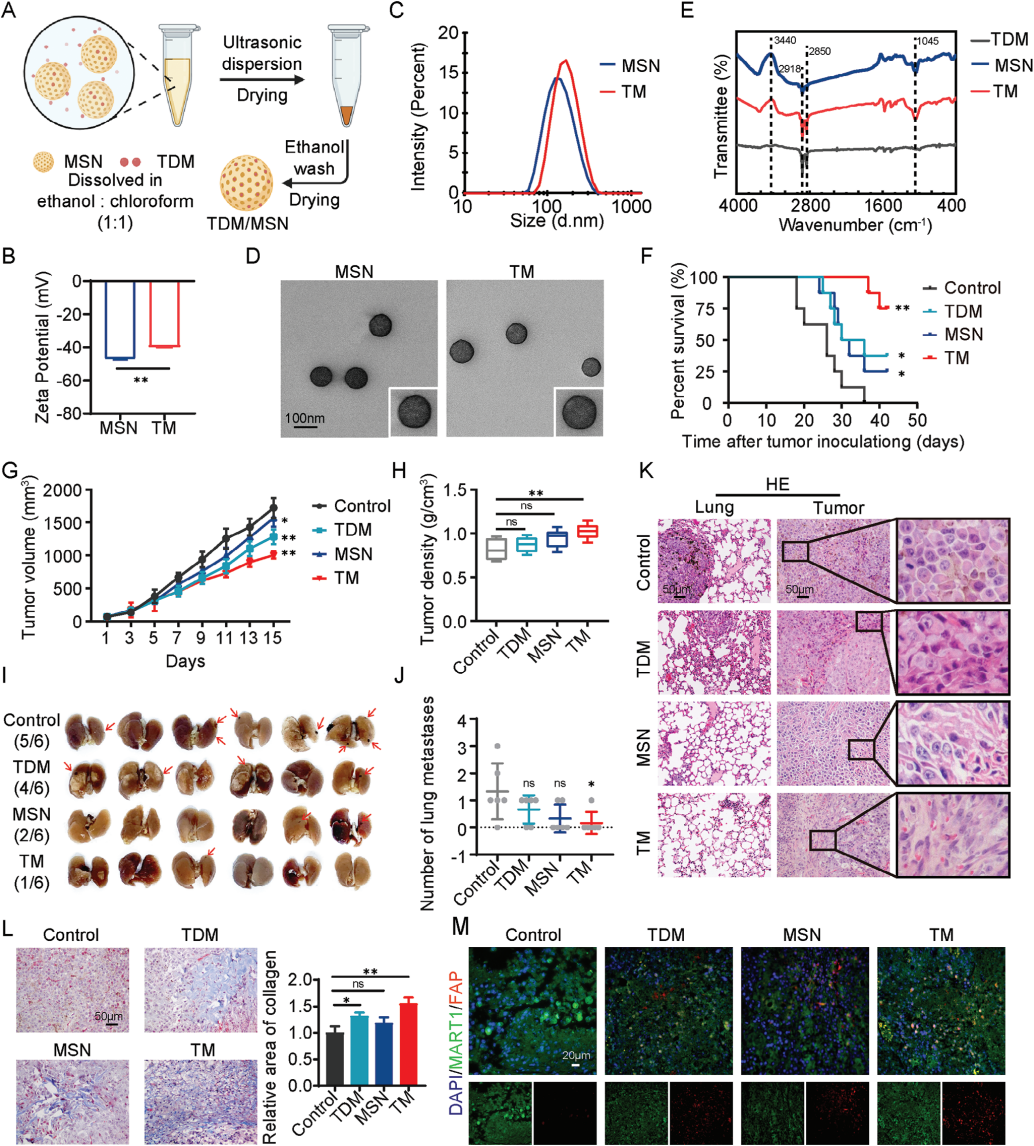
Preparation of TDM/MSN (TM) conjugates and the antitumor activity evaluation. A) Schematic illustration of the procedures for the synthesis of TM. B,C) Zeta potential and particle size of MSN and TM detected by DLS. D) TEM images of MSN and TM. E) FTIR spectra of TDM, MSN, and TM. The peaks on the spectra of TM contained the characteristic peaks of TDM and MSN, indicating the successful loading of TDM into MSN. F,G) Kaplan‐Meier survival curves (F), tumor volume(G), and tumor density(H) of B16‐F10 tumor‐bearing mice administered intratumoral injection of control (saline), TDM, MSN, or TM. I,J) Lung metastases images (I) and numerical statistics (J). The red arrows indicate lung metastatic foci. K) HE staining of lung metastatic foci and tumor. L) Collagen deposition (blue) detected by Masson staining. M) Representative images showing immunofluorescence staining of FAP (red), MART1 (green), and DAPI (blue) in B16 tumor tissue. All values are presented as the mean ± SD, n = 6, ns = not significant, *p < 0.05, **p < 0.01.

Mesoporous silica nanoparticles (MSN) are attractive drug delivery carriers due to their porous structure, excellent biocompatibility, and controllable degradability. The synthesis process of the nanocomposite TDM/MSN (TM) is illustrated in **Figure** **1**A. TDM was successfully loaded in the mesopore channels of the MSNs named TM by electrostatic attraction confirmed by the changes of zeta potentials, which increased by 9.8 mV (Figure 1B). Dynamic light scattering (DLS) analysis revealed that the particle size of MSN was 139.1 ± 5.8 nm, while that of TM was 153.2 ± 1.5 nm (Figure 1C). The loading capacity of TDM on MSN was determined to be 10.2%. The monodispersity and morphology of the nanoparticles have not been altered by a series of synthesis procedures from the transmission electron microscopy (TEM) (Figure 1D). Successful loading of TDM onto MSN was also confirmed by Fourier transform infrared spectroscopy (FTIR) (Figure 1E). The anti‐tumor properties of TM were evaluated using B16‐F10 tumor‐bearing mice, demonstrating a significant improvement in mouse mortality with TM treatment. Compared to the PBS group, tumor growth was inhibited in each administration group, with the TM group exhibiting the highest inhibition rate. This difference became evident after the 9th day of administration (Figure 1F,G). At the treatment endpoint, it was observed that the TM group had the smallest tumor volume but the highest density (Figure 1H). B16‐F10 is a highly metastatic tumor model, and examination of the lungs of tumor‐bearing mice revealed that 83.3% of the control group had tumor metastasis, with some having multiple metastatic foci. However, tumor metastasis in the TM group was significantly inhibited compared to the control group (Figure 1I,J). The HE staining of lung tissue revealed tumor metastasis and pathological changes indicative of fibrosis in the lungs of the TDM group, while the lung alveolar structure was clear and the alveolar walls were thin in the TM group. This observation may be attributed to the enhanced permeability and retention (EPR) effect of MSN loading, which limited the potential damage of direct TDM application to the lungs. The HE staining results of tumor tissue revealed that tumor cells in the control group exhibited characteristics of low differentiation. In the treatment groups, particularly in the TM group, cells showed elongated overall morphology, condensed and flattened nuclei, and deeply stained cytoplasm, displaying features of both tumor cells and fibroblasts, resembling a fibroblastic‐like transformation of tumor cells (Figure 1K). Masson's staining demonstrated collagen deposition accompanying the fibroblastic‐like transformation of cells (Figure 1L). To determine whether this observed fibroblastic‐like transformation was induced by cancer‐associated fibroblasts or by the transformation of tumor cells themselves, we performed tissue immunofluorescence staining. MART1 serves as a marker for melanoma cells, while FAP serves as a marker for activated fibroblasts. The results showed that some cells within treatment group exhibited co‐expression of MART1 and FAP, with this co‐expression being more prominent in the TM group (Figure 1M). In vitro experiments further demonstrated that TM inhibited the viability, migration, and invasion abilities of B16‐F10 cells (Figure S1, Supporting Information). These findings suggest that TM can induce a fibroblast‐like transformation of tumor cells, leading to collagen accumulation and inhibition of tumor growth.

### 2.2 TM Induced Fibroblast‐Like Transformation of Tumor Cells

**Figure 2:**
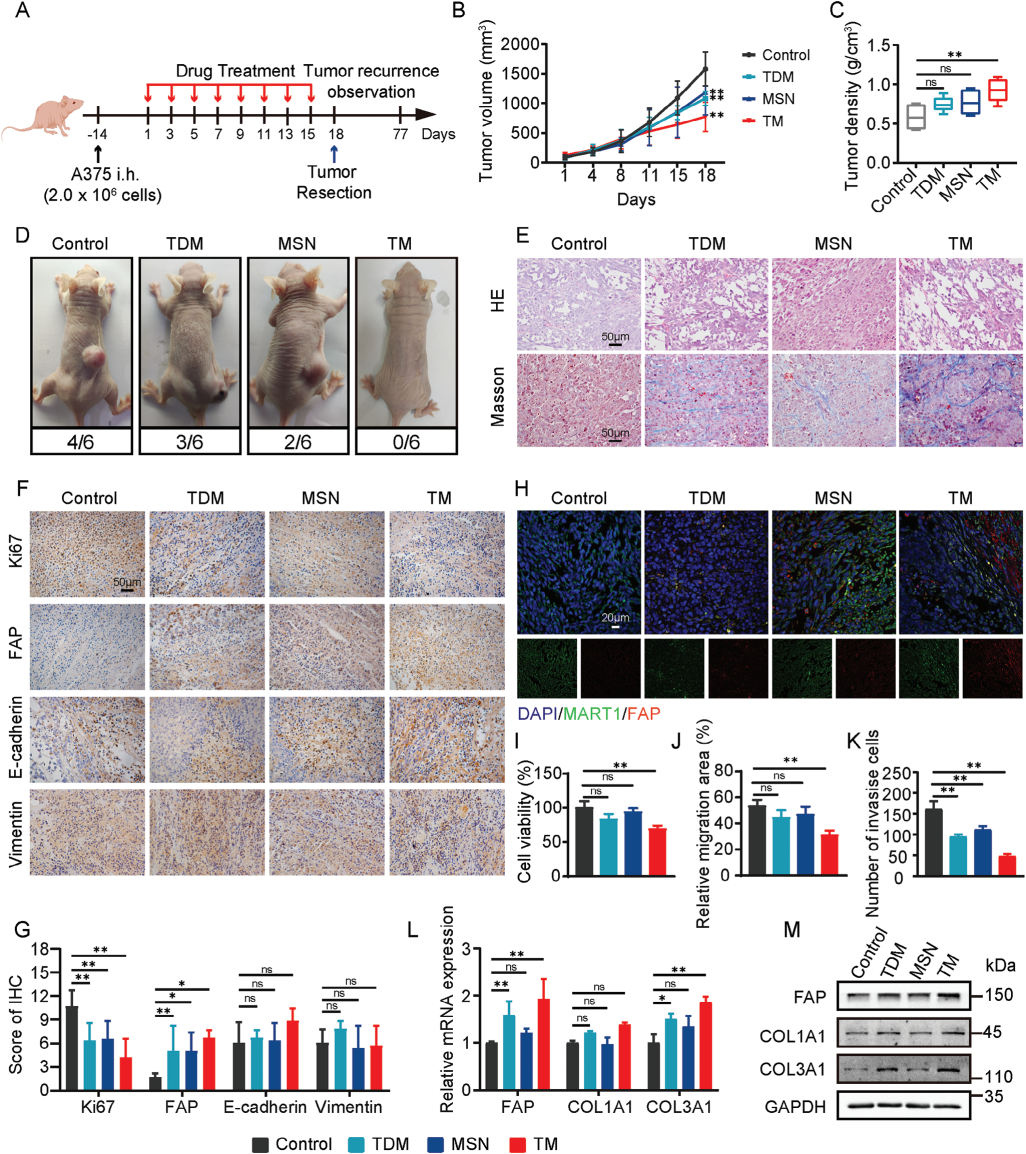
TM induced fibroblast‐like transformation of tumor cells. A) Schematic illustration of drug administration and recurrence monitoring in A375 xenograft tumors. B,C) Tumor volume(B), and tumor density(C) of A375 tumor‐bearing mice administered intratumoral injection of control (saline), TDM, MSN, or TM. D) Representative images and count of recurrent tumors. E) HE and Masson staining of tumor sections. F,G) IHC staining for Ki67, FAP, E‐Cadherin, and Vimentin in the A375 tumor with scoring. H) Representative images showing immunofluorescence staining for FAP (red), MART1 (green), and DAPI (blue) in the A375‐tumor tissue. I) Cell viability was assessed by CCK8 assay. J) Cellular migration was analyzed by cell scratch assay. K) Invasion cells assessed by transwell assay. L,M) Validation of fibrosis‐related genes expression levels by qRT‐PCR (L) and western blot (M). All values are presented as the mean ± SD, n = 6, ns = not significant, *p < 0.05, **p < 0.01.

Current research suggests that tumor fibrosis has profound effects on tumor immunity, especially T cells. To further investigate the possibility of TM‐induced fibroblast‐like transformation of tumor cells to inhibit tumor cell proliferation and metastasis, and whether this process is influenced by T cells, we established an A375 model in nude mice, as shown in **Figure** **2**A. Tumors were removed on the 18th day of drug treatment, simulating clinical surgical resection, and recurrence was observed for 60 days. During the 18‐day treatment, it was observed that the TM group exhibited the best inhibitory effect on tumor growth volume, and the density of excised tumors was significantly increased compared to the control group (Figure 2B,C; Figure S2A, Supporting Information). At the end of the observation for recurrence, the recurrence rates for the control group, TDM group, and MSN group were 66.67%, 50%, and 33.33%, respectively, while no recurrence was observed in the TM group (Figure 2D). HE staining of tumor tissues showed that the tumor cells in the control group were densely arranged with typical characteristics of a high nuclear‐cytoplasmic ratio, while the TM group exhibited numerous nuclear aggregations and deep cytoplasmic staining, with spindle‐shaped cells exhibiting characteristics of both tumor cells and fibroblasts. The results of Mason staining also showed collagen deposition accompanied by fibroblast‐like transformation, which was different from the extracellular matrix deposition of conventional tumor fibrosis. These collagens were deposited inside tumor cells (Figure 2E). To further determine whether the spindle‐shaped transformation is due to TM‐induced Epithelial‐Mesenchymal Transition (EMT) that promotes tumor malignancy, we conducted immunohistochemical (IHC) staining. The results showed that the commonly used markers of EMT, E‐cadherin, and Vimentin, did not exhibit significant changes in expression under the treatments. However, the expression of FAP, representing fibroblast activation, was significantly elevated in all treatment groups compared to the control group. Consistent with the inhibitory effects of treatment on tumor growth, TM significantly reduced the expression of the proliferation marker Ki67 (Figure 2F,G). Further confirmation from immunohistochemical fluorescence staining results indicated that these cells undergoing fibroblast‐like transformation were tumor cells themselves rather than cancer‐associated fibroblasts (Figure 2H). So, is this fibroblast‐like transformation of tumor cells induced by TM beneficial for tumor treatment? We validated this through in vitro experiments, which showed that compared to the control group, TM significantly inhibited the viability of tumor cells (Figure 2I). Scratch and transwell experiments also supported the restrictive effect of TM on the migration and invasion abilities of tumor cells (Figure 2J,K; Figure S2B,C, Supporting Information). Further validation of the fibrotic features of A375 cells was conducted through qRT‐PCR and western blot. The results showed that TM upregulated the expression levels of FAP during in vitro cultivation and primarily led to the accumulation of type III collagen (Figure 2L,M; Figure S2D, Supporting Information). These findings further confirm that TM mainly induces fibroblast‐like transformation of tumor cells, focusing on the accumulation of COL3A1 and progresses into a relatively static fibrosis stage.

### 2.3 EcDNA Influence the Intracellular Collagen Accumulation Induced by TM

**Figure 3:**
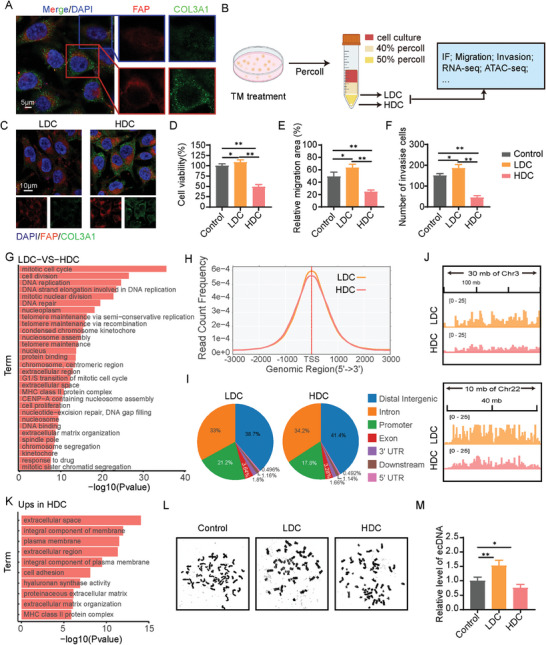
EcDNA influence the intracellular collagen accumulation induced by TM. A) IF of FAP (red), COL3A1 (green), and DAPI (blue) in A375 cells after treatment with TM. B) Schematic illustration of percoll density gradient centrifugation for obtain LDC and HDC. C) IF of FAP (red), COL3A1 (green), and DAPI (blue) in LDC and HDC. D) Cell viability was assessed by CCK8 assay. E) Cellular migration was analyzed by cell scratch assay. F) Invasion cells assessed by transwell assay. G) GO Enrichment analysis of RNA‐seq data. H) Peak distribution curve in the transcription initiation site region from ATAC‐seq. I) Peak statistical map of functional element distribution in the genome from ATAC‐seq. J) Peak are visualized by IGV from ATAC‐seq. K) GO enrichment analysis by combined RNA‐seq and ATAC‐seq. L,M) Representative images (L) and quantification (M) of ecDNA. All values are presented as the mean ± SD, n = 3, ns = not significant, *p < 0.05, **p < 0.01.

Although it has been confirmed that the fibroblast‐like transformation of tumor cells induced by TM can inhibit the malignant progression of tumors, the immunofluorescence (IF) experiments observed that FAP was activated after TM administration. However, it seems that a little fraction of cells did not achieve intracellular accumulation of COL3A1 (**Figure** **3**A). We attempted to explore the functional differences between these two types of cells and whether they affect the therapeutic effect of TM. Based on the difference in collagen accumulation, we separated these two cell types by density using the Percoll method, naming them low‐density cells (LDC) and high‐density cells (HDC), as shown in Figure 3B and Figure S3A (Supporting Information). qRT‐PCR and IF validation confirmed that HDC represented a subset of cells with higher collagen accumulation, while LDC showed a failure in collagen accumulation (Figure S3B, Supporting Information; Figure 3C). We further investigated the functional differences between LDC and HDC. Results from CCK‐8, scratch, and transwell experiments showed that LDC exhibited higher cell viability, migration, and invasion abilities compared to HDC and the control group (Figure 3D–F; Figure S3C,D, Supporting Information). To gain deeper insights into the differences between LDC and HDC and their underlying mechanisms, we conducted RNA‐seq and ATAC‐seq analyses. The Gene Ontology (GO) enrichment analysis of the RNA‐seq results revealed significant pathway differences related to DNA and chromosomes between LDC and HDC (Figure 3G). ATAC‐seq results showed that LDC had higher chromatin accessibility compared to HDC, primarily in the promoter and exon regions (Figure 3H,I). This open peak feature appeared to be patchy, possibly due to the presence of extrachromosomal DNA (ecDNA) (Figure 3J).^[^ ^15^ ^]^ EcDNA is currently considered an important factor in the development of drug resistance. The combined analysis of RNA‐seq and ATAC‐seq also supported the notion that the differential genes between LDC and HDC mainly affect the extracellular matrix and cell adhesion, among other factors (Figure 3K). To validate whether the differences between LDC and HDC are caused by ecDNA, we conducted chromosome smudge analysis. The results showed that LDC indeed had higher levels of ecDNA compared to HDC and the control group (Figure 3L,M). The presence of ecDNA was further confirmed by YoYo‐1 staining (Figure S3E, Supporting Information). These results suggest that the increase in ecDNA may be a significant factor contributing to the generation of LDC.

### 2.4 TMW Induces the Transformation of LDC to HDC by Clearing ecDNA

**Figure 4:**
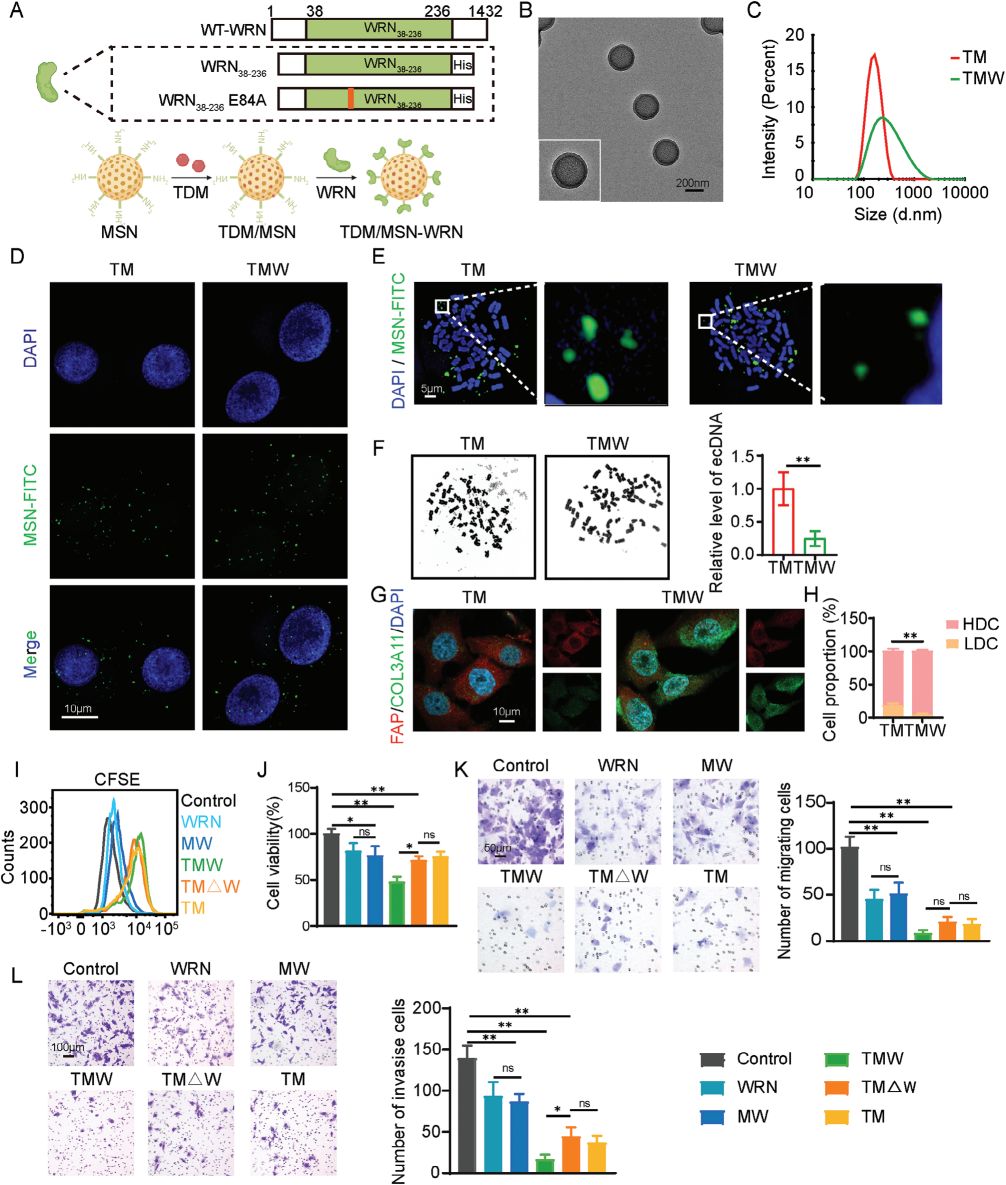
TMW induces the transformation of LDC to HDC by clearing ecDNA. A) Schematic illustration of the construction of WRN_38‐236_ and WRN_38‐236_ E84A with an N‐terminal His tag and the process of conjugation to TM. B) TEM images of TMW. C) Particle size of TM and TMW detected by DLS. D) Representative fluorescent images of LDC uptake TM or TMW by FITC labeled MSN. E) Representative fluorescent images of ecDNA in LDC cleared by TMW. F) Representative images and quantification of ecDNA in LDC after TM or TMW further treatment. G) IF of FAP (red), COL3A1 (green), and DAPI (blue) in LDC after TM or TMW further treatment. H) Proportion of LDC and HDC in A375 cells after administration of TM or TMW. I) Cell proliferation was measured by CFSE staining. J) Cell viability was assessed by CCK8 assay. K,L) Transwell assays were utilized to assess cell migratory without matrigel (K) and invasive ability (L). All values are presented as the mean ± SD, n = 3, ns = not significant, *p < 0.05, **p < 0.01.

Due to the potential negative impact of LDC on the anti‐tumor effect of TM, we modified a highly efficient Dnase onto the nanoparticles TM to clear ecDNA and force the transformation of LDC into inactive HDC. WRN is a unique member of the Dnase family, possessing both helicase and nuclease activities. However, due to its large size with 1432 amino acids and relative instability, we selectively expressed and purified the truncated form WRN_38‐236_, retaining enzymatic activity, as well as the mutated protein WRN_38‐236_E84A (△WRN_38‐236_) as a control.^[^ ^16^ ^]^ The specific preparation method of the nanoparticles is depicted as shown in **Figure** **4**A. After TDM were loaded in the mesopore channels of the MSNs, WRN_38‐236_/△WRN_38‐236_ was linked onto the outside surface of the MSN by an EDC/NHS‐mediated covalent coupling method named TDM/MSN‐WRN (TMW). TEM observed TMW monodisperse spherical nanoparticles with protein surface coating (Figure 4B). The DLS results showed that the particle size of TMW was 256.7 ± 7.3 nm with uniform dispersion, which was significantly larger than TM (Figure 4C; Figure S3A, Supporting Information). We tested that WRN exhibits enzymatic activity, whereas △WRN lost its enzymatic activity due to E84A mutation (Figure S4B, Supporting Information). The stability of TMW was found to be well‐maintained when stored at −4 °C for 14 days (Figure S4C, Supporting Information).

We selectively isolated LDC to analyze whether TMW could help inhibit this subset of cells that responded poorly to TM. Firstly, the uptake of nanoparticles by LDC was evaluated in vitro using FITC‐modified MSN. The results indicated that both TM and TMW were efficiently taken up by the cells, and there was no significant difference in the amount of uptake (Figure 4D; Figure S4D, Supporting Information). This uptake was inhibited by sucrose, indicating that the process is mediated by clathrin‐dependent endocytosis (Figure S4E, Supporting Information). Further fluorescence examination of chromosome smudge revealed the distribution of ecDNA around MSN in the LDC of the TM group, possibly due to hydrogen bonding between Si‐OH and ecDNA. However, these ecDNA can be cleared under the action of WRN in TMW (Figure 4E). Broader statistical analysis results demonstrate that TMW can clear 75.11% of ecDNA in LDC (Figure 4F). Moreover, IF results indicate that after TMW cleared ecDNA in LDC, they exhibited intracellular collagen accumulation similar to HDC (Figure 4G). Through qRT‐PCR, it was revealed that collagen accumulation under TMW treatment became significant at 72 hours post‐administration (Figure S4F, Supporting Information). After culturing A375 cells in vitro and treating them with TM or TMW, Percoll gradient centrifugation was performed to separate LDC and HDC, followed by cell counting. The results showed that after TM treatment, the proportion of LDC was 17.896±3.876%, while after TMW treatment, the proportion of LDC decreased to 4.485±2.007%. This supports the result that TMW helps reduce the proportion of LDC, potentially leading to a better anti‐tumor effect. To investigate further, we conducted cell functional experiments. CFSE‐labeled cells were analyzed by flow cytometry, revealing that the TMW group exhibited the most significant inhibition of tumor cell proliferation. The results of cell viability detection by CCK8 also supported the above conclusion (Figure 4I,J). Cell cycle analysis revealed arrest at the G2 phase after treatment with TMW (Figure S4G, Supporting Information). Additionally, migration and transwell assays demonstrated that TMW exerted the best inhibitory effect on the migration and invasion potential of A375 cells among all groups (Figure 4K,L). The comparison of cellular functional differences between the TMW and TM△W groups confirm the enhancement of the anti‐tumor effect of TM by WRN. The above results show that TMW can reduce the proportion of LDC by clearing ecDNA to achieve a more comprehensive induction of fibroblast‐like transformation in tumor cells, and the addition of WRN helps TMW show a better anti‐tumor effect than TM.

### 2.5 MMP‐Carrying ecDNA Affects Type III Collagen Accumulation in Tumor Cells

**Figure 5:**
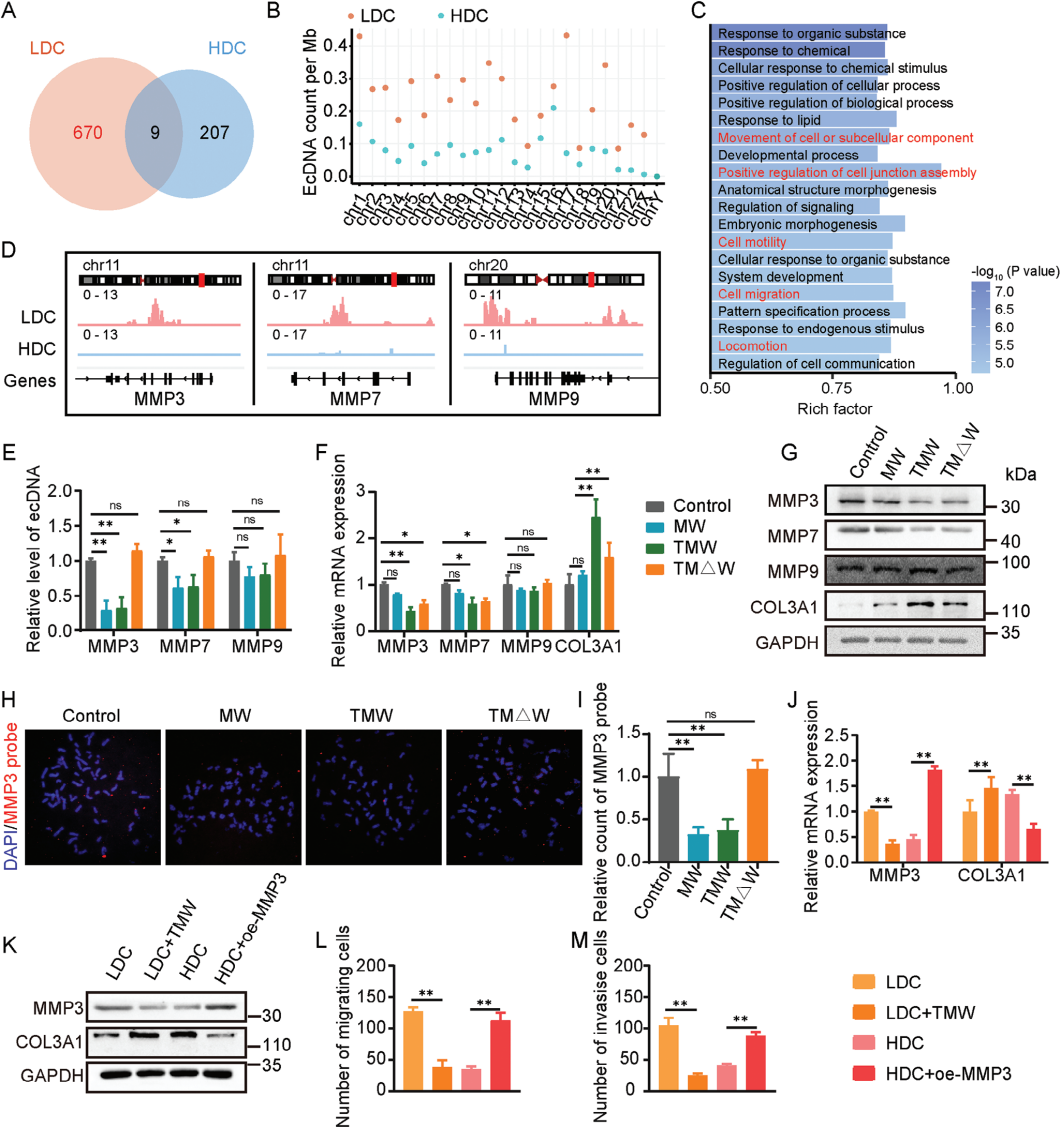
MMP‐carrying ecDNA affects type III collagen accumulation in tumor cells. A) Venn plot of ecDNA in LDC and HDC. B) EcDNA distribution on chromosomes. C) Gene annotation and GO analysis of the different ecDNA in the LDC. D) Cricle‐seq peaks of MMP3, MMP7, and MMP9 in LDC and HDC. E) qRT‐PCR analysis of MMP3, MMP7, and MMP9 levels in purified ecDNA. F) qRT‐PCR analysis of MMP3, MMP7, MMP9 and COL3A1 mRNA. G) Western blot analysis of MMP3, MMP7, MMP9, and COL3A1 levels. H,I) Representative images of MMP3‐carrying ecDNA in A375 cells were identified by Fish probe and quantitatively analyzed. J,K) Validation of MMP3 and COL3A1 expression levels by qRT‐PCR (J) and western blot (K). L,M) Transwell assays were utilized to assess cell migratory without matrigel (L) and invasive ability (M) in LDC, LDC+TMW, HDC, and HDC+oe‐MMP3. All values are presented as the mean ± SD, n = 3, ns = not significant, *p < 0.05, **p < 0.01.

To gain further insights into the differences in ecDNA abundance and function between LDC and HDC, we conducted circl‐seq. The results indicated that LDC indeed exhibited a higher abundance of ecDNA compared to HDC, and this difference was observed across all chromosomes (**Figure** **5**A,B; Figure S5A, Supporting Information). Moreover, ecDNA larger than 10 kb may potentially contain full gene sequences (Figure S5B, Supporting Information). Through gene annotation and enrichment analysis of the differential ecDNA present in LDC, we found that these ecDNA mainly affected cell motility and migration functions (Figure 5C). Among them, the MMP family members MMP3, MMP7, and MMP9, which are closely associated with collagen accumulation, showed significantly broader distribution of circle‐seq peaks in LDC compared to HDC (Figure 5D). To validate the presence of ecDNA carrying MMP family genes, we purified ecDNA from A375 cells treated with drugs and performed relative quantification using qRT‐PCR. The results showed a reduction in ecDNA carrying MMP3, MMP7, and MMP9 under the action of WRN (Figure 5E). However, the mRNA levels of cells after drug treatment in each group did not entirely correlate with the levels of ecDNA, suggesting a co‐regulation by genomic DNA and ecDNA (Figure 5F). Western Blot results showed that the protein translation level was consistent with the mRNA level (Figure 5G; Figure S5D, Supporting Information). Since MMP3 is the most significantly changed gene, having a significant impact on patient prognosis, and is an enzyme directly related to COL3A1, we used MMP3 as a representative for further investigation (Figure S5C, Supporting Information). We employed MMP3 fluorescence in situ hybridization (FISH) probe labeling and observed that in the Control and TM△W groups, numerous FISH probe signals were scattered across the chromosomes, supporting the existence of ecDNA carrying MMP3. The MW and TMW groups exhibited significantly reduced FISH signals, likely due to the action of WRN in the nanomaterial (Figure 5H,I). We explored further whether these MMP3‐carrying ecDNAs affected the accumulation of collagen in LDCs and their functional differences from HDCs. Qp and Western Blot results revealed that MMP3 levels were higher in LDC compared to HDC. Under the influence of TMW, this difference could be eliminated, concurrently aiding in the accumulation of collagen in LDC. Additionally, overexpression of MMP3 in HDC inhibited the accumulation of COL3A1 (Figure 5J,K; Figure S5E, Supporting Information). Correspondingly, after TMW treatment, LDC showed inhibited migration and invasion abilities. However, overexpression of MMP3 restored the migration and invasion abilities of HDC (Figure 5L,M; Figure S5F,G, Supporting Information). These findings suggest that TMW, by clearing ecDNA carrying MMP3, which is crucial for affecting collagen accumulation in LDC, enhances the anti‐tumor effect. These results indicate that TMW eliminates the resistance of LDCs to fibrosis induction by clearing MMP3‐carrying ecDNA that affects LDC collagen accumulation, and overall improves the anti‐tumor effect.

### 2.6 TMW Suppresses Tumor Malignancy Progression by Inducing Tumor Fibrosis in PDX Models

**Figure 6:**
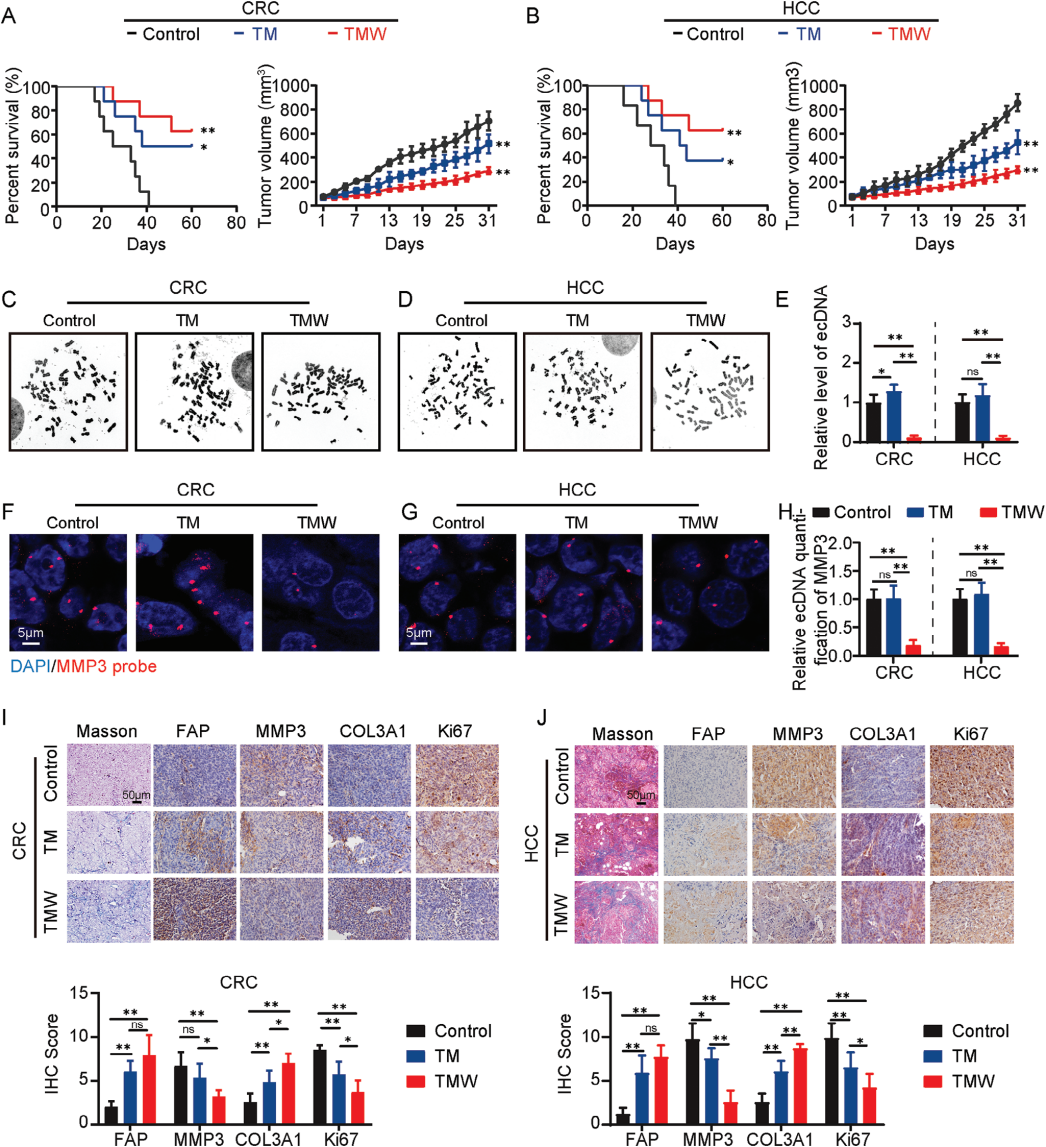
TMW suppresses tumor malignancy progression by inducing tumor fibrosis in PDX models. A) Survival and tumor volume curves of PDX model of CRC after treated with control (saline), TM, or TMW. B) Survival and tumor volume curves of PDX model of HCC after treated with control (saline), TM, or TMW. C–E) Representative images and quantification of ecDNA from cells isolated from the PDX model. F–H) Representative images of MMP3‐carrying ecDNA in cells isolated from the PDX model were identified by Fish probe and quantitatively analyzed. I,J) Representative images of Msaaon and IHC staining for FAP, MMP3, COL3A1, and Ki67 in the CRC PDX model and scored. K,L) Representative images of Msaaon and IHC staining for FAP, MMP3, COL3A1, and Ki67 in the HCC PDX model and scored. All values are presented as the mean ± SD, n = 6, ns = not significant, *p < 0.05, **p < 0.01.

TMW has shown a better effect on inhibiting the malignant progression of tumors than TM at the cellular level. We first tested the safety of the drug in C57BL/6 mice, and the results showed that during drug treatment, there were no significant changes in the body weight of mice compared to the control group, and blood biochemical tests indicated no impairment of liver and kidney function in the mice due to the drug (Figure S6A,B, Supporting Information). Further support for the safety of drug treatment was provided by HE staining of various organs (Figure S6C, Supporting Information). Subsequently, we explored the clinical application potential of TMW using patient‐derived xenograft (PDX) models (Figure S6D, Supporting Information). To broaden the potential clinical applications, we expanded our research to investigate the therapeutic potential of TMW in colorectal cancer (CRC) and hepatocellular carcinoma (HCC). The PDX model demonstrates that TMW extends survival, suppresses tumor volume, and increases tumor density in both CRC and HCC (**Figure** **6**A,B; Figure S6E, Supporting Information). Furthermore, statistical analysis of chromosomal smears reveals a significant increase in ecDNA levels in the TM group compared to the control group in CRC, while in HCC, there is no significant difference in ecDNA levels between the TM group and the control group, possibly due to the relatively low proportion of LDC in the total cell population, which did not lead to overall statistical differences. Nevertheless, TMW effectively eliminates ecDNA in both CRC and HCC (Figure 6C–E). We further conducted FISH analysis to examine whether the PDX model also exists ecDNA with MMP3. The results were consistent with the overall changes in ecDNA levels. The TMW treatment group exhibited a significant reduction in FISH signals compared to the control and TM groups (Figure 6F–H). Masson and IHC results showed further accumulation of collagen, especially COL3A1, after TMW administration, while the proliferation‐related malignant marker Ki67 was significantly inhibited. At the same time, through in‐depth analysis of the IHC results, it was found that the increased expression of FAP may be mainly affected by TDM and MSN, while the changes in MMP3 are affected by both the genome and ecDNA (Figure 6I,J). The above results confirm that TMW can enhance the positive effect of inhibiting the malignant progression of tumors by clearing ecDNA, whether in CRC or HCC.

## 3 Discussion

From a physiological perspective, tissue repair relies on parenchymal repair and fibrosis repair.^[^ ^17^ ^]^ In tumor tissue, self‐repair primarily involves parenchymal repair (filled by cancer cell proliferation), while the body's scar repair is an ancient ability. Inducing tumors to switch repair modes may organize tumors and exert anticancer effects. Many types of tumors such as pancreatic cancer, breast cancer, and prostate cancer exist fibrosis. Therapeutic interventions such as radiotherapy and chemotherapy may further induce tumor fibrosis.^[^ ^18^ ^]^ Current research on tumor fibrosis primarily focuses on CAFs. However, there is still debate regarding whether fibrosis acts as a promoting or inhibiting factor in cancer. Some studies report that fibrosis supports cancer growth through various mechanisms, including direct cell interactions, immune modulation, and ECM remodeling. On the other hand, there is substantial evidence suggesting that tumor‐associated fibrosis can inhibit cancer proliferation and metastasis. Clinical studies correlating the expression of type I collagen and CAFs markers such as αSMA and FAP with disease outcomes indicate that patients with high stromal proliferation may have improved prognosis and overall survival in pancreatic cancer, breast cancer, and lung cancer.^[^ ^19^ ^]^ However, due to the lack of specificity of CAF markers, the definition of CAF subpopulations remains unclear, and it cannot be ruled out that they may originate from the tumor itself. This study primarily focuses on whether tumor parenchymal cells themselves can undergo a similar fibroblast‐like transformation and whether this transformation inhibits cancer. This is entirely distinct from the current concept of tumor fibrosis. Exploring whether inducing tumor cells to undergo fibroblast‐like transformation could serve as a potential anti‐cancer therapy deserves further investigation.

This study developed a novel nanoparticle TM to induce a population expressing both tumor cell and fibroblast markers simultaneously, exhibiting spindle‐shaped morphology and accumulating collagen similar to fibroblasts. This fibroblast‐like transformation of tumor cells is distinct from EMT that promotes tumor malignancy, as the related markers E‐cadherin and Vimentin showed no significant changes after TM treatment. Typically, tumor EMT leads to enhanced tumor cell migration, invasion, and poorer prognosis, contrary to the changes observed after TM treatment. We used two factors that induce tissue fibrosis to superimpose the induction of foreign body granuloma and inflammatory granuloma in tumor cells, revealing for the first time that tumor cells may also undergo a fibroblast‐like transformation in which collagen accumulates internally. Moreover, type III collagen derived from cancer cells exerted an unexpected anti‐tumor effect, entirely consistent with scar formation characteristics, which is different from past research on CAFs. Some potential mechanisms include: 1) excessive deposition of collagen in cancer cells binds organelles or excessively consumes energy, limiting other energy‐consuming activities. 2) Local deposition of cancer cell‐derived collagen selectively changes the environment around cancer cells. Such local deposition could have mechanical or biochemical effects on cancer cells. 3) Various post‐translational modifications and the assembly of collagen in cancer cells induced by TM/TMW are special, thus endowing cancer cell‐derived collagen with different functions.^[^ ^20^ ^]^ This needs further research and may lead to a new therapy after tumor immunotherapy: mobilizes the original scar ability of the body.

According to reports, TDM can exert anti‐tumor effects by activating immune cells such as dendritic cells and macrophages.^[^ ^9^ ^]^ In addition, TDM can also induce immune responses similar to Mycobacterium tuberculosis (MTB) infection, including the production of pro‐inflammatory cytokines and the formation of inflammatory granulomas. In this process, the recognition of TDM by the receptor Mincle is crucial.^[^ ^21^ ^]^ Silica can induce models of silicosis, representing the process of foreign body granuloma formation. Some studies suggest that silicosis is caused by oxidative stress induced by silica, while others propose that it is due to the release of various bioactive substances such as TNF‐α and TGF‐β triggered by free radical chain reactions.^[^ ^22^ ^]^ Whether inflammatory granuloma or foreign body granuloma, the persistence will develop into tissue organization characterized by an increase in collagen fibers, with fibroblasts gradually decreasing and transforming into a quiescent state, and eventually into fibrotic tissue. Using MSN to load TDM can achieve efficient drug delivery, and superimpose the dual induction effects of inflammatory granuloma and foreign body granuloma, enhance the potential of inducing tumor fibrosis, and is expected to inhibit the malignant progression by promoting organized tumors.

This study also found that some tumor cells achieve resistance to fibrosis induction through ecDNA under stressful conditions. Sequencing results showed that these ecDNA carry coding sequences for matrix metalloproteinases (MMPs), which maintain the balance between ECM accumulation and its degradation. MMPs promote tumor progression and metastasis by degrading ECM components, which enhances cell migration and invasion.^[^ ^23^ ^]^ In the absence of MMPs, ECM degradation may be impaired, leading to excessive accumulation of matrix proteins and promoting fibrosis development.^[^ ^24^ ^]^ This suggests that the main driving force for tumor parenchymal repair may come from the active presence of ecDNA in tumor cells. WRN, a broad‐spectrum human DNA helicase, is unique among all RecQ helicases because of N‐terminal 3′ to 5′ exonuclease activity. EcDNA can be cleared with the assistance of WRN, but the reasons for the generation of these ecDNAs remain unclear. Whether they are pre‐existing or induced by selection requires further investigation. We speculate that genomic damage may activate the defense mechanisms of some cancer cells, leading to the formation of ecDNA through broken genomic DNA looping, consistent with Paul's findings. This is also an important direction for further research. The oncogenes encoded on ecDNA are liberated from chromosomal constraints, allowing for rapid self‐replication and transcription of numerous associated genes. This enables tumors to swiftly alter their genomes in response to ever‐changing environments, accelerating tumor evolution and leading to treatment resistance. This also suggests that future cancer therapies may need to pay more attention to the impact of tumor evolution from ecDNA.

In conclusion, this study proposes a new anti‐tumor strategy. MSN‐loaded TDM can effectively inhibit tumor growth and metastasis, combined with surgical resection after administration can reduce the possibility of tumor recurrence. During the treatment, nanoparticles can enhance retention in tumor tissues through the EPR effect to reduce the systemic side effects of TDM. Further research found that most tumor cells can achieve internal accumulation of type III collagen with the characteristics of fibrous cells under the induction of TM. This phenomenon represents a tumor cell‐intrinsic solidification distinct from the tumor microenvironment solidification caused by CAFs. However, IF experiments revealed that despite TM inducing high expression of FAP, there were still a small number of cells that did not fully accumulate collagen in accordance with fibrosis characteristics. These cells were named LDC and exhibited higher cellular activity, migration, and invasive capabilities. Further research found that the resistance of LDC to TM‐induced fibrosis was mediated by an increase in ecDNA levels. To eliminate the impact of LDC on the overall anti‐tumor efficacy, we further conjugated WRN onto TM. Subsequent research confirmed that TMW could effectively eliminate ecDNA, exerting a better anti‐tumor effect. In summary, TMW represents a promising therapeutic approach by inducing tumor cell fibroblast‐like transformation to inhibit malignant progressions such as tumor growth and metastasis. However, our study relied on existing methods of inducing tissue fibrosis, suggesting the potential for developing more efficient inducers specifically targeting tumor fibrosis. Moreover, while our investigation provided valuable insights, further depth is required in future studies to elucidate the mechanisms underlying the effects on the tumor microenvironment and the processes involved in ecDNA generation.

## 4 Experimental Section

### Cell Culture

Cells were purchased from KeyGen Biotech. B16‐F10 cells and A375 cells were cultured in 1640 medium containing 10% FBS. TDM (3 µg mL^−1^), MSN (30 µg mL^−1^), TM (30 µg mL^−1^, according to the weight of MSN) were applied for in vitro administration respectively.

### Mouse Tumor Bearing Model

C57BL/6 (6 weeks) and BALB/c Nude mice (3 weeks) were purchased from Charles River. B16‐F10 cells (1.0 × 10^6^ cells) and A375 cells (2.0 × 10^6^ cells) were respectively injected subcutaneously into the back of C57BL/6 mice and BALB/c nude mice. When the average tumor volume reached 200 mm^3^, the mice were randomly divided into four experimental groups: Control; TDM (1 mg kg^−1^); MSN (10 mg kg^−1^); TM (10 mg kg^−1^, according to the weight of MSN). Each group received drug injections every two days, and tumor volumes were recorded. The mice bearing A375 tumors were surgically removed on the 18th day and observed for tumor recurrence. These mice were fed a normal diet and maintained in pathogen‐free conditions with a 12‐hour light/dark cycle. All animal experiments were carried out by the Guide for the Care and Use of Laboratory Animals published by the US National Institutes of Health under the approval of the Animal Ethics Committee (2021‐SYDWLL‐000480).

### H&E Staining

The mice were euthanized, and the tissues were fixed in 10% formalin, embedded in paraffin, and cut into 4 µm sections. The tissue sections were then baked at 60 °C for 4–6 hours before staining. After deparaffinization and hydration of the tissue sections, they were stained with hematoxylin for 3 min, followed by rinsing with running water for 1 min. Subsequently, the sections were differentiated in 1% hydrochloric acid‐ethanol solution and blued with 0.6% mild ammonia water. After staining with eosin for 30 s and rinsing with running water for 30 s, the sections were dehydrated with ethanol, transparent with xylene, and finally sealed with neutral resin.

### MASSON Staining

The tissue sections were impregnated into a mordant solution at room temperature overnight and then washed with water for 10 min. According to the kit instruction, these sections were stained with Celestite Blue Solution for 3 min, Mayer Hematoxylin Solution for 3 min, differentiated by Acid Differentiation Solution for 5 seconds, dropped by Ponceau‐Acid Fuchsin Solution for 10 min. These sections were washed after each dyeing step. Next, these sections were treated with Phosphomolybdic Acid Solution for 10 min and Aniline Blue Solution for 5 min, and rinsed in Acetic Acid Solution for 2 min. Finally, tissue sections were dehydrated by alcohol, transparent by xylene, and sealed with neutral resin for analysis.

### Immunohistochemistry (IHC) Staining

Paraffin‐embedded tumor tissue sections were performed with Sodium citrate antigen retrieval solution (Solarbio) and blocked with goat serum. After washing with 1xPBS and distilled water, the sections were incubated with primary antibodies against Ki67 (Affinity, USA), FAP (PTMab, China), E‐Cadherin (Cell Signaling Technology, USA), Vimentin (Affinity, USA), MMP3 (Santa, USA) and COL3A1 (Affinity, USA) at 4 °C overnight. After incubating with secondary antibody, these sections were stained with DAB and hematoxylin dye solution and sealed with neutral resin (Solarbio, China) finally. The IHC scores were assessed separately by three experts.

### Migration and Invasion Assays

Transwell migration and invasion assays were performed using a transwell plate (Corning, USA) coated with (for invasion) or without (for migration) Matrigel (Corning, USA). 200 µL of serum‐free medium (2 × 10^5^ cells) was added to the upper chamber and 500 µL of medium containing 10% FBS was added to the lower chamber. Cells were incubated at 37 °C for 24–48 hours. The cells (and Matrigel) inside the chamber were used a cotton swab to wipe off and fixed at room temperature for 10 min using 4% paraformaldehyde (PFA) (Solarbio, China). The chamber was stained with crystal violet solution (Beyotime, China) for 10 min, rinsed with PBS and left to dry, and finally photographed under a microscope (Leica, Germany)

### Quantitative Real‐Time PCR (qRT‐PCR)

RNA was extracted using MolPure Cell RNA Kit (Yeasen, China), and then RNA reverse‐transcribed into cDNA using Hifair III 1st Strand cDNA Synthesis SuperMix (Yeasen, China) according to manufacturer's instructions. Gene expression was determined using primers specific for each gene on a Real‐time fluorescence quantitative PCR instrument (Bio‐Rad, CFX touch 96, USA) using 2× HQ SYBR (Zomanbio, China). GAPDH was used as the internal control and the result was analyzed via the 2^−ΔΔCt^ method.

### Western Blot

Cells were lysed with RIPA lysis buffer on ice. The lysates were centrifuged at 12 000 rpm for 10 min at 4 °C and collecting cell supernatant was quantified using BCA kit (Thermo Scientific, USA). Identical quantities of proteins were separated by sodium dodecyl sulfate‐polyacrylamide gel electrophoresis (SDS‐PAGE) and transferred onto PVDF membranes. After blocking with 5% skim milk, incubated with primary antibodies against COL3A1 (1:1000, Affinity, USA), COL1A1 (1:1000, Affinity, USA), MMP3 (1:1000, Proteintech, China), MMP7 (1:1000, Cell Signaling Technology, USA), MMP9 (1:1000, Affinity, USA), FAP (1:1000, Affinity, USA), GAPDH (1:10 000, Affinity, USA) at 4 °C overnight. After incubating with horseradish peroxidase (HPR)‐conjugated secondary antibody at room temperature for 2 h, detection was performed using western blot imaging system.

### Immunofluorescence (IF) Staining

Cells fixed with 4% PFA (Solarbio, China) and permeabilized with Blocking Buffer (Beyotime, China) at room temperature for 20 min. The cells were incubated with the primary antibodies against FAP (1:200, Affinity, USA) and COL3A1 (1:200, Affinity, USA) at 4 °C overnight, followed by incubation with fluorescein‐conjugated secondary antibody. The samples were imaged using a Zeiss LSM800 confocal microscope.

### Preparation of TM and TMW

MSN‐NH_2_ and MSN‐FITC were purchased from XFnano. For the preparation of TDM/MSN (TM), TDM (5 mg) and MSN (10 mg) were dispersed in 1 mL solution of ethanol: chloroform (1:1) in a 1.5 mL Eppendorf tube. After ultrasonic dispersion and rotation in the dark for 24 hours, TM was obtained by centrifugation, washed with ethanol, and dried at room temperature. WRN_38‐236_ or WRN_38‐236_E84A was covalently conjugated onto MSN‐NH_2_ through the amine group using cross‐linking reagents EDC and NHS. Specifically, 1 mg of TM was dissolved in 1 mL MES buffer (pH 5.6), followed by the addition of 5 mg of EDC and 5 mg of NHS. The mixture was stirred at room temperature for 2 hours. Subsequently, 10 µL of WRN solution (20 µg mL^−1^) was added to the above solution, and the mixture was stirred for another 2 hours at room temperature. Excess EDC, NHS, and WRN were removed by repeatedly washing the nanoparticles with PBS several times before lyophilization.^[^ ^25^ ^]^ Further characterization confirmed the success of the nanomaterial synthesis.

### Chromosome Spread Test

Cells in logarithmic growth phase were induced with colchicine (0.2 µg mL^−1^) for 3 hours. The cell suspension was collected in a 15 mL centrifuge tube, adding 5 mL hypotonic solution (0.075 m KCL) drop by drop and maintaining hypotonicity in a 37 °C water bath for 30 min. The tubes were shaken immediately after adding 1 mL of ice‐cold fixative (methanol: glacial acetic acid = 3:1) and centrifuged at 1000 g for 10 min. The cell pellets were fixed 1–2 times using 5 mL of ice‐cold fixative depending on the number of cell pellets. Finally, cells were resuspended in fresh fixative and dropped vertically onto moist cold glass slides, and then sealing the slide with DAPI. Images were acquired by the ZEISS LSM800.

### Percoll

Percoll and 1.5 m NaCL (9:1) were prepared into an isotonic percoll solution (SIP). Fill 1 mL of 50% Percoll (0.15 m NaCL: SIP = 1:1), 1 mL of 40% Percoll (0.15 m NaCl: SIP = 2:3) and 1 mL of cell suspension into a 15 mL centrifuge tube from bottom to top, centrifuge at 400 g for 25 min. Cells in 40% (top) and 50% (bottom) percoll layer were collected and named low density cells (LDC) and high density cells (HDC), respectively, washed with ice‐cold PBS to remove percoll particles. LDC and HDC were counted, and their viability was assessed using trypan blue. Finally, LDC and HDC were continuously cultured for later use.

### Extraction of ecDNA

The cells were collected in pre‐cooled PBS. After centrifugation at 10 000 rpm for 20 min at 4 °C, the resulting cell pellets were resuspended thoroughly in pre‐cooled solution I (25 mm Tris‐HCl (pH 8.0), 10 mm EDTA, 50 mm Glucose), and added an equal volume of solution II (250 mm NaOH, 1% SDS) and gently inverted several times. After 40–60 s at room temperature, the solution was immediately added 1.5 times the volume of solution III (3 m potassium acetate, 5 m acetic acid) and kept in ice bath for 10 min. The cell supernatant was collected at 12 000 rpm, 4 °C, 10 min, and added an equal volume of solution IV (phenol: chloroform: isoamyl alcohol = 25: 24: 1) at room temperature for 20 min. After another centrifugation, the supernatant was added an equal volume of solution V (chloroform: isoamylol = 24:1) at room temperature for 10 min, repeating this step. The precipitate was washed with absolute ethanol and 70% ethanol, and finally using 50 µL of sterile, enzyme‐free water to elute the DNA. The obtained DNA is treated with Plasmid‐Safe exonuclease to digest linear DNA.

### Protein Purification

WRN construct (residues 38–236 aa) was appended with an N‐terminal His_6_ tag and was cloned into a pET28b vector (Genscript, China). WRN_38‐236_ was overexpressed in E. coli Rosetta (DE3) cells (Solarbio, China) by IPTG (0.2 mm) induction. Cells were lysed and sonicated on ice. After centrifugation at 10 000 g for 30 min at 4 °C and then incubated with the equilibrated His‐tag Purification Resin Column (His‐tag Protein Purification Kit, Beyotime) overnight at 4 °C. The flow‐through was collected, followed by washes with Nondenaturing Wash Buffer, then eluted with Nondenaturing Elution Buffer. Finally, the collected eluent was concentrated using a 10 kDa ultrafiltration tube (Millipore, USA). Mutant (WRN_38‐236_E84A) was constructed, expressed and purified using the same protocol^[^ ^16^ ^]^

### Patient‐Derived Xenograft (PDX) Tumor Model

Fresh tumor tissues obtained from cancer patients were cut into small cubes measuring 3 × 3 × 3 mm and implanted into 4‐week Nog mice. When the tumors reached a volume of 800 mm^3^, the mice were euthanized, and the tumor tissues were collected and designated as F1. The same procedure was repeated to obtain F2. Ultimately, mice carrying F3 transplant tumors were used for drug treatment experiments. The mice were randomly divided into three experimental groups: Control (Normal saline); TM; TMW (10 mg kg^−1^, according to the weight of MSN). Each group received drug injections every two days, and tumor volumes were recorded. All experiments were carried out under the approval of the Medical Ethics Committee of Tianjin Medical University General Hospital (IRB2024‐YX‐326‐01).

### Fluorescent In Situ Hybridization (FISH)

The tissues and cells were conducted utilizing Fluorescent in situ hybridization. Tumor tissue sections were deparaffinized with xylene and serially rehydrated with 100%, 90%, 80%, and 70% ethanol, then washed with 1× PBS for 5 min. Cell slide was dried at 75 °C for 3 h. The prepared sample slide was soaked in 0.1% NP‐40/2 × SSC for 30 min, followed by permeabilization with 50 µL digestive working solution (protease K: 1 × TBS = 1:100) for 10 min at 37 °C and dehydration with graded ethanol. The slide was added 40 µL of denaturing solution, and then the slide and Probe hybridization mixture (1 µL probe with 39 µL FISH Hybridization Buffer) were denatured at 88 °C for 8 min respectively. Then the Probe hybridization mixture was transferred to ice for 8 min and the slide was dehydrated by graded ethanol. Covered with the prewarmed coverslip, the slides were incubated in a hybridization furnace at 43 °C in the dark overnight for 24–48 h. Next, Coverslip was removed and slides washed with the prewarmed 2 × SSC at 53 °C for 5 min, 0.1% NP‐40/2 × SSC at 42 °C for 5 min, and 2 × SSC at 42 °C for 5 min. After washing and drying, the slide was stained with DAPI and imaged using a Zeiss LSM800 confocal microscope.

### Statistical Analysis

Statistical analyses were performed using GraphPad Prism 8, and the results were expressed as mean ± SD. Differences between groups were assessed through unpaired two‐tailed *t‐*test (for two‐sample comparison) or one‐way ANOVA with Dunnett's test (for multiple comparisons). Statistical significance was set at *P < 0.05 or **P < 0.01.

## Conflict of Interest

The authors declare no conflict of interest.

## Author Contributions

Y.L., X.H., and Y.L. contributed equally to this work. T.S. and H.L. designed this study. T.S., H.L., and Y.L. wrote, reviewed, and revised the manuscript. Y.L., X.H., Y.L., Q.Q., C.C., and Y.C. performed the experiments, analyzed, and interpreted data. T.S. and W.Z. provided administrative, technical, or material support. All authors read and approved the final manuscript.

## Supporting information

Supporting Information

## Data Availability

The data that support the findings of this study are available from the corresponding author upon reasonable request.
