# Supplementary material for: WRN Nuclease‐Mediated EcDNA Clearance Enhances Antitumor Therapy in Conjunction with Trehalose Dimycolate/Mesoporous Silica Nanoparticles
Source: Adv Sci (Weinh). 2024 Aug 29;11(40):2407026. doi: 10.1002/advs.202407026 (PMC11516056; doi:10.1002/advs.202407026)
Supplement: Supplementary file 1 — Supporting Information [file ADVS-11-2407026-s001.docx]

**Supporting Information for**

**WRN Nuclease-Mediated EcDNA Clearance Enhances Antitumor Therapy in Conjunction with Trehalose Dimycolate/Mesoporous Silica Nanoparticles**

Yinan Li^1,#^, Xiu Huang^1,3,#^, Yingying Li^1,3,#^, Qingqing Qiao^1^, Caihong Chen^1^, Yang Chen^1^, Weilong Zhong^2,*^, Huijuan Liu^1,3,*^, Tao Sun^1,*^

^1^State Key Laboratory of Medicinal Chemical Biology and College of Pharmacy, Nankai University, Tianjin, China.

^2^Tianjin Key Laboratory of Digestive Diseases, Department of Gastroenterology and Hepatology, Tianjin Institute of Digestive Diseases, Tianjin Medical University General Hospital, Tianjin, China.

^3^Tianjin Key Laboratory of Molecular Drug Research, Tianjin International Joint Academy of Biomedicine, Tianjin, China.

^#^These authors contributed equally to this work as co-first authors.

^*^Corresponding author Email: tao.sun@nankai.edu.cn (T.S.); liuhuijuanxyz@163.com (H.L.); [zhongweilong@tmu.edu.cn](mailto:zhongweilong@tmu.edu.cn) (W.Z.)

**
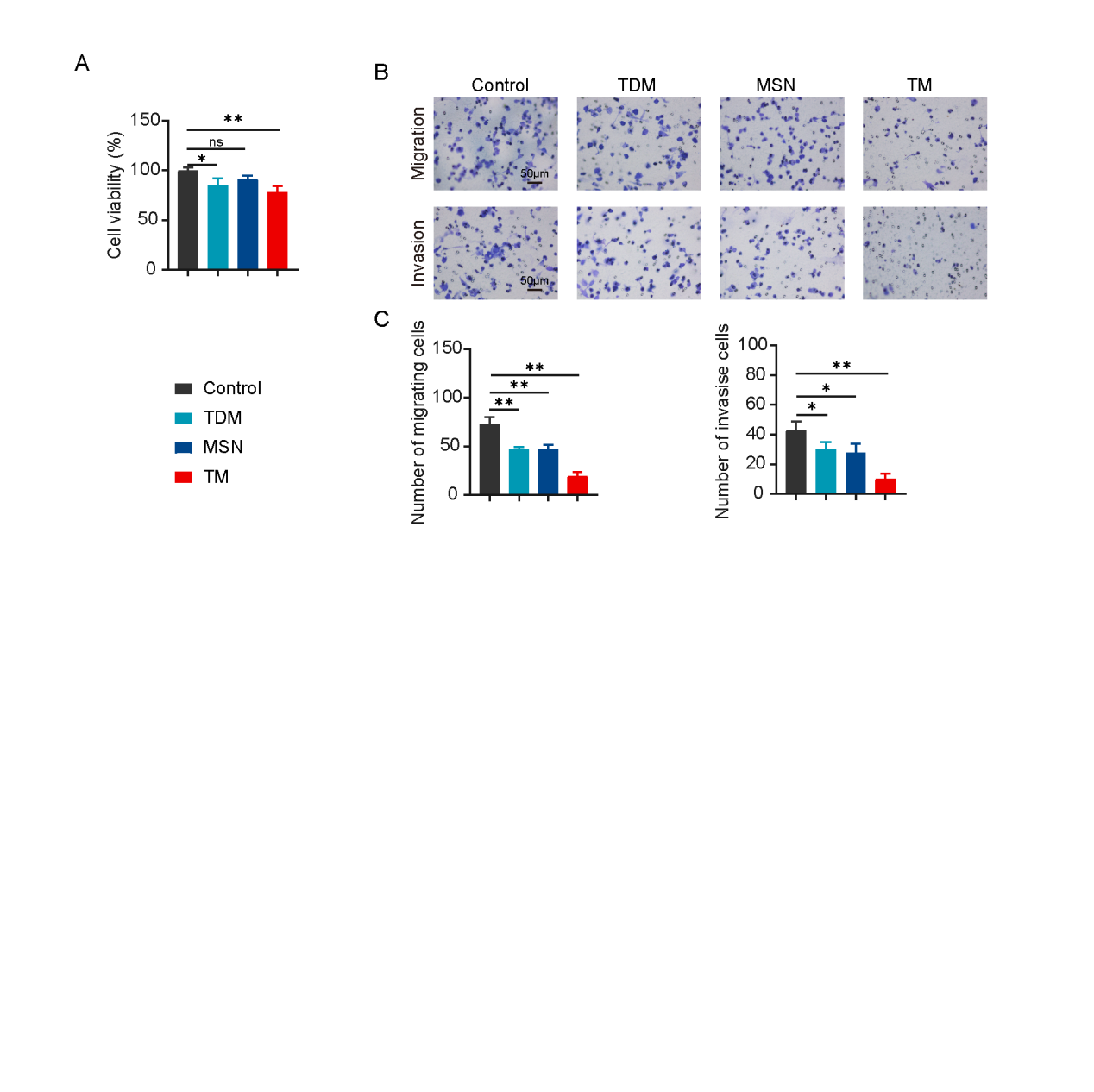
**

**Figure S1.** A) Cell viability was assessed by CCK8 assay. B-C) Transwell assays were utilized to assess cell migratory without matrigel and invasive ability.


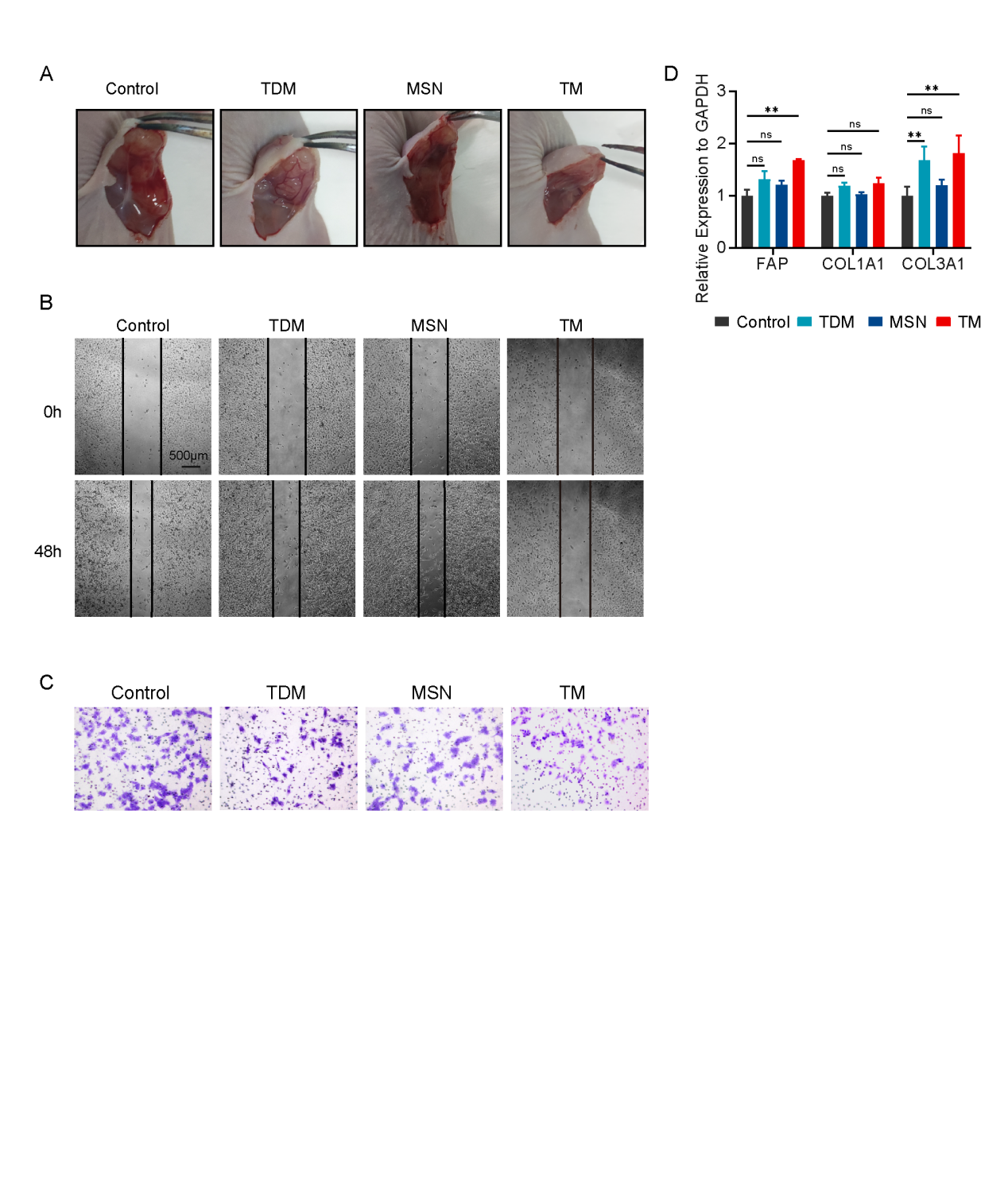


**Figure S2.** A) Surgical excision of the tumor. B) Cellular migration was analyzed by cell scratch assay. C) Invasion cells assessed by transwell assay. D) Grayscale analysis results for FAP, COL1A1 and COL3A1.

**
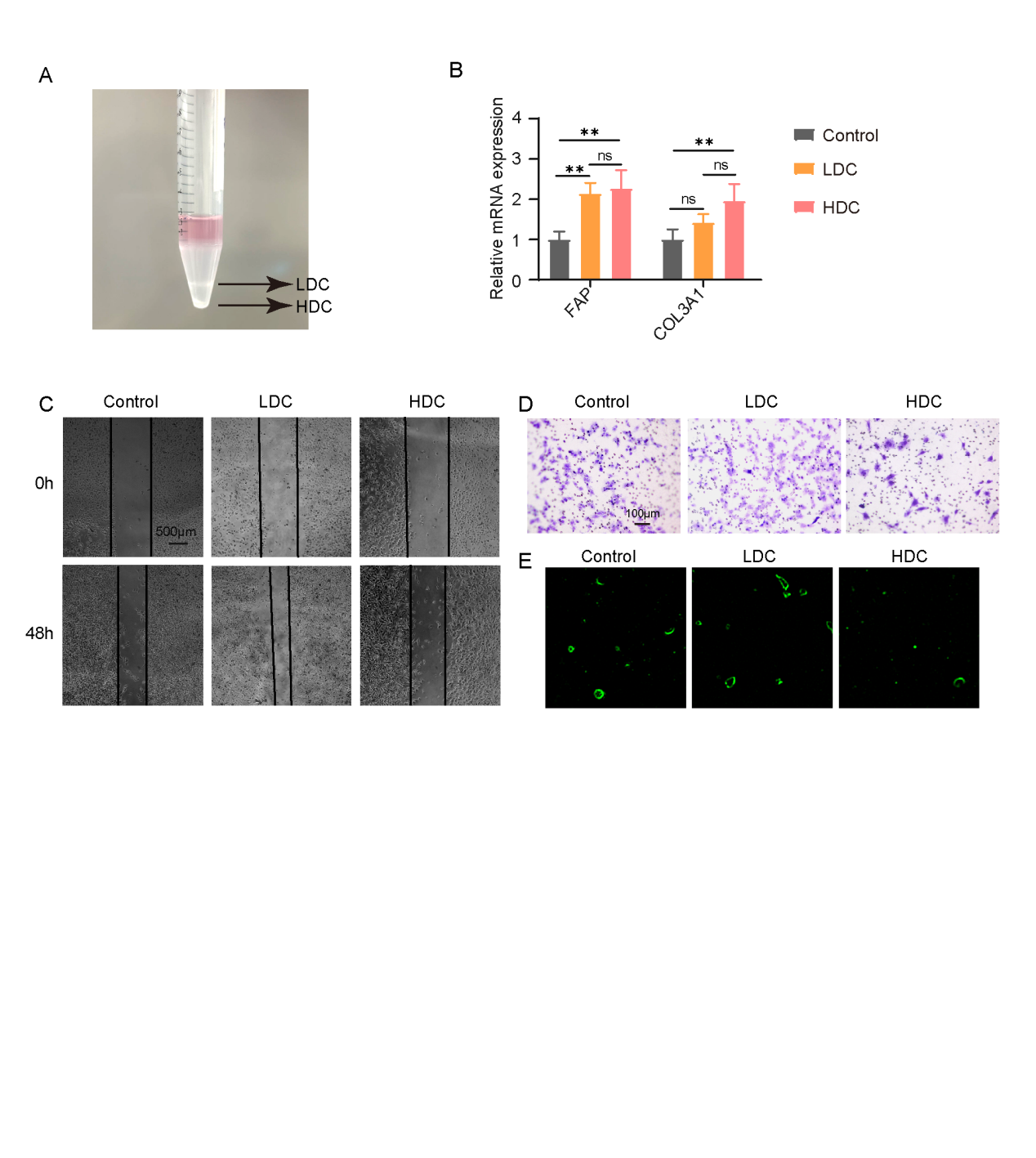
**

**Figure S3.** A) Percoll [density gradient](https://www.sciencedirect.com/topics/biochemistry-genetics-and-molecular-biology/density-gradient) centrifugation for obtain LDC and HDC. B) qPCR analysis of FAP and COL3A1 mRNA. C) Cellular migration was analyzed by cell scratch assay. D) Invasion cells assessed by transwell assay. E) Fluorescence image of ecDNA stained by YoYo-1.


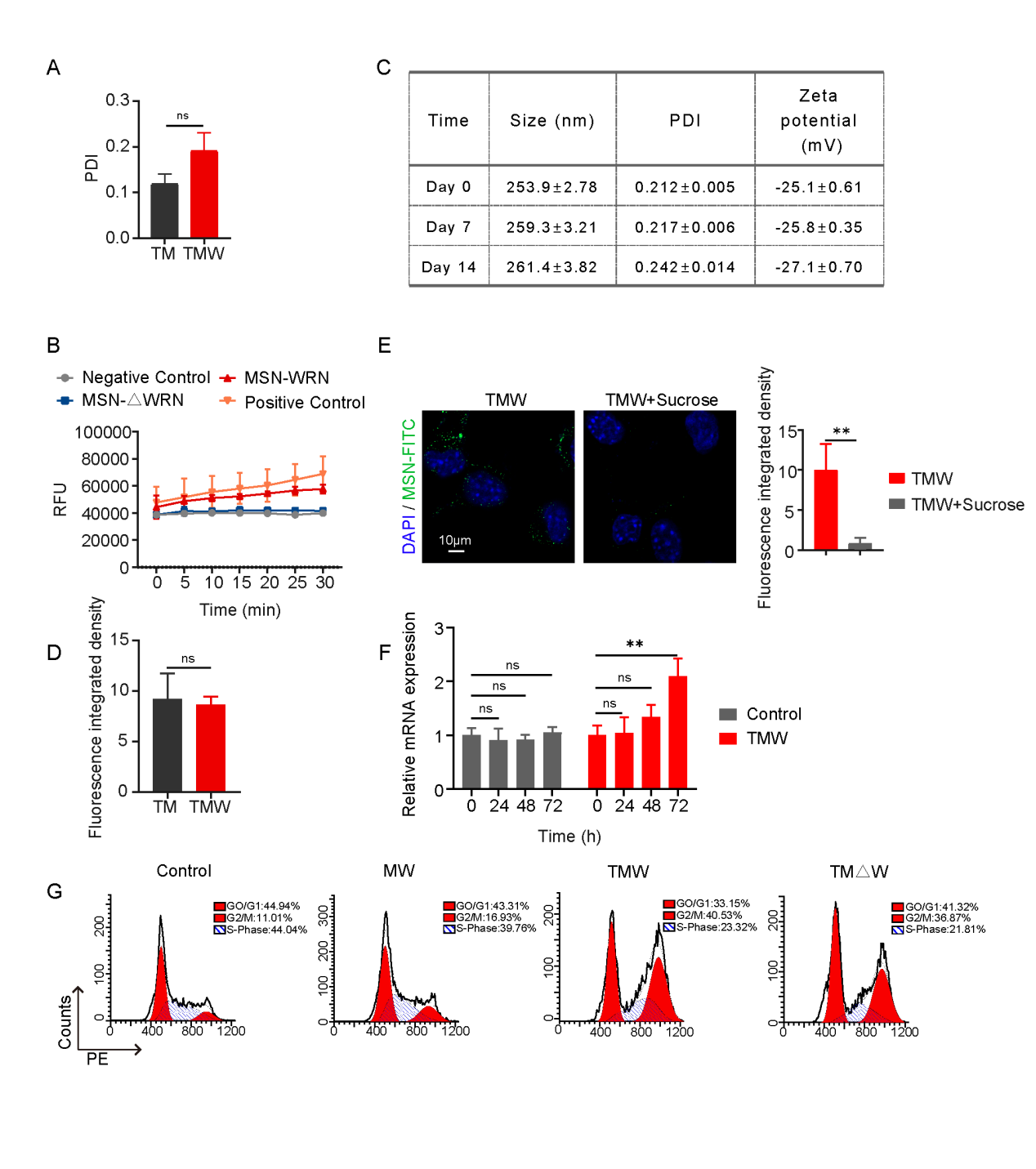


**Figure S4.** A) PDI of TM and TMW. B) DNase activity measured using DNase activity fluorescence detection system. C) Stability characterization of TMW nanoparticles using Dynamic Light Scattering. D) Fluorescence quantification of LDC uptake of FITC-labeled nanoparticles. E) Sucrose inhibits the uptake of TMW by cells. F) qRT-PCR analysis of COL3A1 after treated with TMW. G) Flow cytometry detect the cell cycle.

**
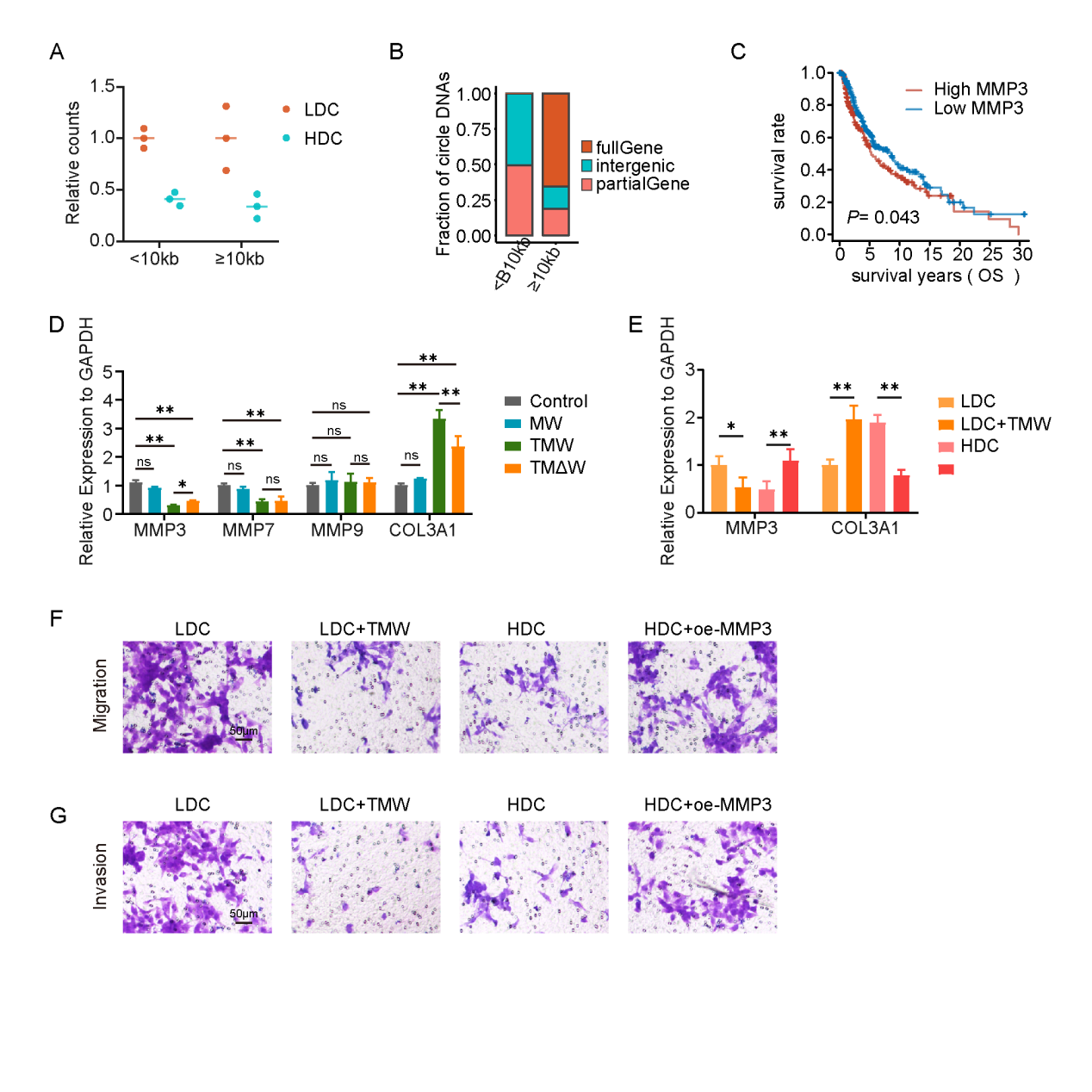
**

**Figure S5.** A) EcDNA quantity statistics chart. B) Statistical diagram of the relationship between EcDNA and complete gene inclusion. C) MMP3-related survival analysis in melanoma from TCGA. D) Grayscale analysis results for MMP3, MMP7, MMP9 and COL3A1. E) Grayscale analysis results for MMP3 and COL3A1. F-G) Transwell assays were utilized to assess cell migratory without matrigel (F) and invasive ability (G).

**
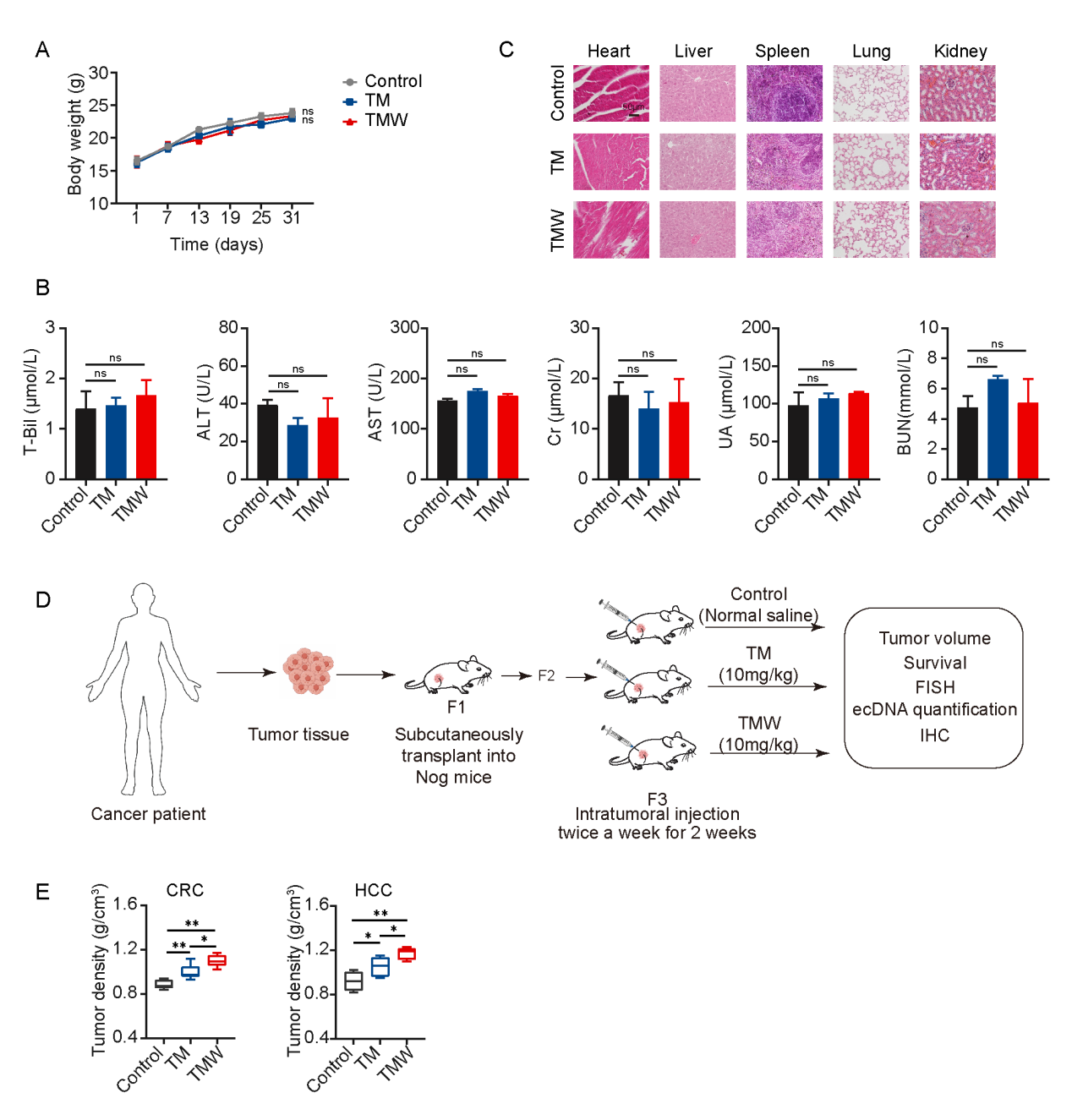
**

**Figure S6.** A) Changes in body weight of mice in each group after administration. B) Blood biochemistry indicates the liver (T-bil, ALT, AST) and kidney (Cr, UA, BUN) functions of mice in each group after administration. C) HE of the organs of mice in each group after administration. D) Schematic of the experimental workflow of PDX. E) Tumor density of PDX model of CRC and HCC after treated with control (saline), TM, or TMW.
